# Deconjugation of Polychlorinated Biphenyl Sulfates to Hydroxylated PCBs by Anaerobically Cultured Mouse and Human Gut Microbiota

**DOI:** 10.1021/acs.chemrestox.5c00016

**Published:** 2025-03-25

**Authors:** Xueshu Li, Joe J. Lim, Cayen Rong, Hans-Joachim Lehmler, Julia Yue Cui

**Affiliations:** † Department of Occupational and Environmental Health, University of Iowa, Iowa City, Iowa 52242, United States; ‡ Department of Environmental and Occupational Health Sciences, University of Washington, Seattle, Washington 98105, United States

## Abstract

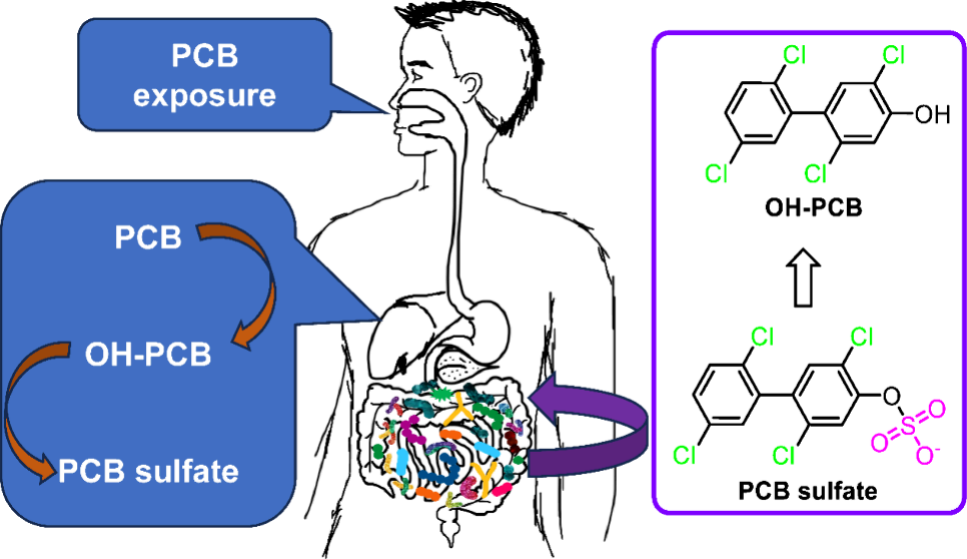

The role of the gut microbiome in metabolizing polychlorinated biphenyls (PCBs), toxic environmental contaminants, and their metabolites remains unclear. This study used mouse and human microbiomes in anaerobic cultures to investigate the metabolism of PCB sulfate to hydroxylated PCBs (OH-PCBs). All microbiomes enzymatically hydrolyzed PCB sulfates. Higher chlorinated PCB sulfates were metabolized more readily. Male mouse microbiomes exhibited more PCB sulfate hydrolysis to OH-PCBs than female mouse microbiomes. Human microbiomes metabolized PCB sulfates to a more considerable extent than mouse microbiomes. They also showed variability in PCB sulfate metabolism, depending on the microbial communities. These findings suggest that the microbiome contributes to PCB metabolism.

Polychlorinated biphenyls (PCBs) are persistent organic pollutants originating from legacy pollution and their inadvertent production as byproducts in manufacturing processes such as paint pigments. PCB metabolism plays a significant role in their toxicity.^1^ Cytochrome P450 enzymes metabolize PCBs to hydroxylated PCBs (OH-PCBs). Further metabolism of OH-PCBs includes the formation of PCB sulfates by cytosolic sulfotransferases. Recent studies demonstrate that PCB sulfates are metabolites formed in PCB-exposed laboratory animals and are present in serum from humans. Although the sulfation of OH-PCBs was initially considered a detoxification process, growing evidence suggests that PCB sulfates are also toxic. This toxicity may arise from direct interactions of PCB sulfates with receptors or through their conversion by mammalian sulfatases back into OH-PCBs. This cycling between PCB sulfates and OH-PCBs can contribute to toxic effects, such as endocrine disruption, neurotoxicity, and other toxicities linked to PCB exposure.

PCB sulfates are excreted into the gastrointestinal content of laboratory animals.^1^ The gastrointestinal tract is home to the microbiome, a diverse community of millions of microbes that play a role in health and disease. The microbiome metabolizes xenobiotics into more readily bioavailable metabolites potentially toxic to the host. Growing evidence suggests that differences in the microbiome explain, in part, inter-individual differences in drug metabolism.^2^ Sulfatases make up a diverse group of enzymes expressed by the microbiome. Metabolism by these enzymes is pivotal in metabolizing various endogenous and exogenous compounds including drugs, nutrients, and other xenobiotics. Analogous to mammalian sulfatases, microbial sulfatases likely influence the bioavailability, activity, and detoxification of these compounds. However, it is unknown if microbial sulfatases metabolize PCB sulfates, major metabolites in the gastrointestinal tract.^1^ Moreover, structure–metabolism relationships for microbial deconjugation reactions have not been established for PCB sulfates. Finally, it is unknown how the large variability of the human microbiome affects the deconjugation of PCB sulfates to OH-PCBs that may be reabsorbed in the gastrointestinal tract, thus extending the OH-PCB half-life and potentially increasing their toxicity to the host.

This pilot study provides initial insights into these unanswered questions by exploring the metabolism of a mixture of four structurally diverse PCB sulfates, including PCB3 sulfate, PCB11 sulfate, PCB25 sulfate, and PCB52 sulfate, to OH-PCBs by anaerobic mouse and human microbial cultures. These PCB sulfates were selected because they, or the corresponding hydroxylated PCBs (OH-PCBs), are human-relevant metabolites (Tables S1 and S2). Because the metabolism of the parent PCBs depends on the degree of chlorination, with metabolism decreasing with an increasing degree of chlorination, the PCB sulfates selected for this study also differ in their degree of chlorination, ranging from 1 to 4 chlorine substituents. To study the microbial metabolism of these PCB sulfates, DMSO (vehicle) or the PCB sulfate mixture (1 mg/mL, 0.71 mM each, total 2.83 mM) in DMSO, sterilized by filtering with 0.22 μm filters, was incubated in 1 mL of a microbial suspension (50 mg intestinal content/mL). Microbial suspensions were obtained from male and female conventional C57Bl/6 mice; see the Supporting Information. The intestinal content from germ-free mice was used as a negative control. In addition, two human microbial communities were used to determine whether the variability in the microbial abundance, determined by metagenomic shotgun sequencing (Figure 1), affects the metabolism of PCB sulfates. The human stool samples from de-identified healthy male (community 1) and female subjects (community 2) were purchased from BioIVT (Westbury, NY, USA) and pooled *n* = 2 per sex. After incubation at 37 °C for 6 h, incubation mixtures were quenched with an equal volume of 1% formic acid, 0.1% l-cysteine, and 0.0001% resazurin in deoxygenated DPBS.

**Figure 1:**
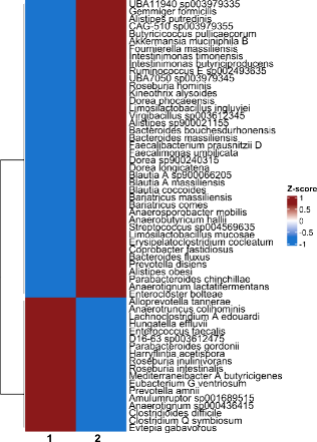
Distribution of microbial abundance between two human microbial communities. Metagenomic shotgun sequencing was performed before incubation; see the Supporting Information. Red and blue colors show at least 30% higher or lower abundance between the two human samples at the species level.

PCB metabolites were extracted from 10 μL of the supernatants by adding 40 μL of Milli-Q water, 10 μL of internal standard solution (3-F,4′-OH-PCB3 and 4-F,4′-PCB3 sulfate, 1 μg/mL each in methanol, Table S1), 90 μL of methanol, and 100 μL of acetonitrile. The mixtures were vortexed and kept in a −20 °C freezer for 30 min. The samples were vortexed and centrifuged at 19,083*g* at 4 °C for 10 min. The supernatants were passed through hybrid phospholipid solid-phase extraction (HybridSPE) cartridges (1 mL, Millipore Sigma, Burlington, MA, USA). The HybridSPE cartridges were washed with 800 μL of methanol. The eluents were combined and kept at −20 °C. The pellets were resuspended in 0.5 mL of Milli-Q water, transferred to 2 mL of microcentrifuge tubes with 100 μL of stainless-steel beads (0.2 mm diameter, Next Advance Inc., NY, USA), and homogenized with a Bullet Blender Storm 24 instrument (Next Advance Inc., NY, USA). The homogenates were centrifuged for 10 min at 19,083*g* and 4 °C. The supernatant was transferred to a 2 mL microcentrifuge tube and acidified with 0.5 mL of Milli-Q water with 1% formic acid. Ten μL of these homogenates were cleaned up as described for the supernatants above.

OH-PCB and PCB sulfate levels in the supernatant and pellet extracts were analyzed on an Agilent 1290 Infinity II UPLC system (Agilent Technologies, Inc. Headquarters, Santa Clara, CA, USA) equipped with an Acquity UPLC-BEH-C18 column (particle size 1.7 μm, inner diameter 2.1 mm, length 100 mm, Waters, Milford, MA, USA), and coupled with a SCIEX Triple Quad 7500 system (SCIEX, Framingham, MA, USA). The flow rate of the mobile phase was 0.3 mL/min. The mobile phase was (A) Milli-Q water with 10 mM triethylammonium acetate and (B) methanol. The following solvent gradient (% B) was used: 0–1.0 min 60%, 1.0–9.5 min 60%, 9.5–12.0 min 60–99%, and 12.0–15.0 min 60%. The temperature of the column chamber was 30 °C. The analysis was performed in the negative multiple reaction monitoring (MRM) mode (Table S3) with a spray voltage of 4500 V. The gas pressure of ion source 1, ion source 2, and curtain gas were 35, 70, and 40 psi, respectively. The source temperature was 450 °C. The sample injection volume was 2 μL. OH-PCBs and PCB sulfates were quantified based on matrix-matched calibration curves and expressed on a molar percentage of the PCB sulfate–OH-PCB pairs in the supernatant and pellet.

PCB sulfates were predominantly present in the supernatant across all experimental conditions, with only 9–20% found in the pellet (Figure 2, Table S4). This distribution aligns with previous in silico and cell culture studies showing that PCB sulfates partition primarily into the aqueous phase. OH-PCBs were also present in the supernatant but in much smaller proportions (0.4–28%), with even lower levels in the pellet (<12%). Incubations with conventional microbiomes (M_CV_ and F_CV_) contained higher levels of OH-PCBs than incubations using intestinal contents from germ-free mice, consistent with enzymatic hydrolysis of PCB sulfates by microbes. The formation of OH-PCBs in control incubations with intestinal content from germ-free mice can attributed to the chemical hydrolysis of PCB sulfates in aqueous solutions.^3^

**Figure 2:**
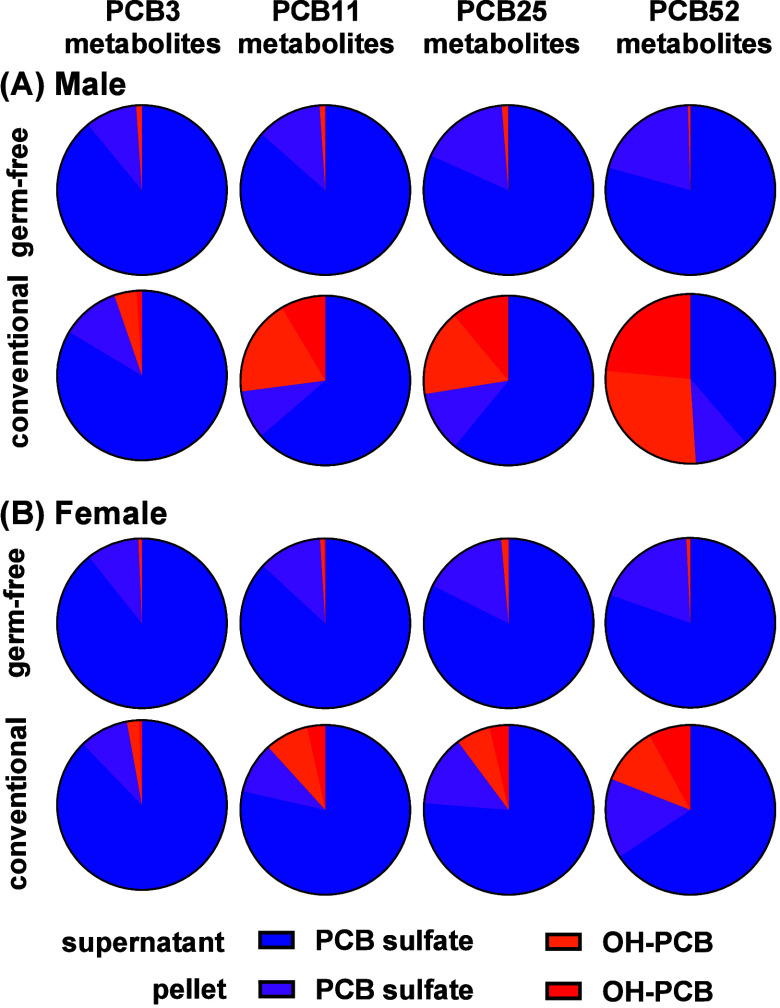
Molar percentage profiles of PCB metabolites (PCB sulfate and corresponding OH-PCB) in the media and pellet after the PCB sulfates were incubated with the microbiome from (A) male and (B) female germ-free and conventional mice.

Sex differences in microbiome metabolism were evident in the mass profiles (Figure 2, Table S4). PCB sulfates consistently showed higher percentages in the supernatant of F_CV_ compared to M_CV_ incubations. In contrast, the percentages in the pellet were similar. M_CV_ microbiome incubations contained higher percentages of OH-PCBs in both fractions, particularly for OH-PCB11 and OH-PCB52, where percentages in M_CV_ were more than double those in F_CV_ incubations. These findings suggest a greater microbial capacity for PCB sulfate metabolism in male microbiomes. These results are consistent with the well-documented sex differences in the composition and function of the gut microbiome in mice.^4^

Chlorination also influenced PCB metabolite profiles (Figure 2, Tables S4–S6). As chlorination increased, the supernatant percentages and supernatant-to-pellet (S/P) ratios of PCB sulfates decreased, with conventional microbiomes showing more pronounced reductions for highly chlorinated congeners like PCB52. Conversely, OH-PCB levels increased in the supernatant with higher chlorination, especially in conventional microbiomes, but their S/P ratios decreased, indicating a greater pellet association for more chlorinated OH-PCBs. PCB52 sulfate exhibited the highest biotransformation, while PCB3 sulfate showed the least. These trends reflect the interplay between lipophilicity and metabolism in the incubations, with higher chlorinated congeners being more readily transformed and associated with microbial activity.

The human microbiome expresses xenobiotic processing genes with considerable inter-individual variability.^5^ Therefore, the enzyme-catalyzed hydrolysis of PCB sulfates was investigated in incubations with two different human microbiome communities. The results from human microbiome experiments reveal distinct community-dependent differences in the metabolism of PCB sulfates to OH-PCBs (Figure 3, Table S7). PCB sulfates were predominantly present in the supernatant across all experimental conditions, with higher percentages observed in the incubations with community 2 than community 1 microbiome (e.g., 65% vs 38% of PCB3 sulfate, respectively). Conversely, OH-PCBs were more abundant in the supernatant and pellet fractions of experiments using the community 1 microbiome, reflecting a greater capacity for the microbial transformation of PCB sulfates to OH-PCBs. For example, OH-PCB3 accounted for 35% of the supernatant fraction in incubations with microbial community 1 but only 8% in the experiments using microbial community 2. Analogous to the experiments with mouse microbiomes, higher chlorinated congeners (e.g., PCB52 metabolites) were more strongly associated with the pellet fraction across both communities, consistent with an increased lipophilicity. Moreover, incubations with human microbiomes showed a more pronounced metabolism of PCB sulfates to OH-PCBs than the experiments with mouse microbiomes.

**Figure 3:**
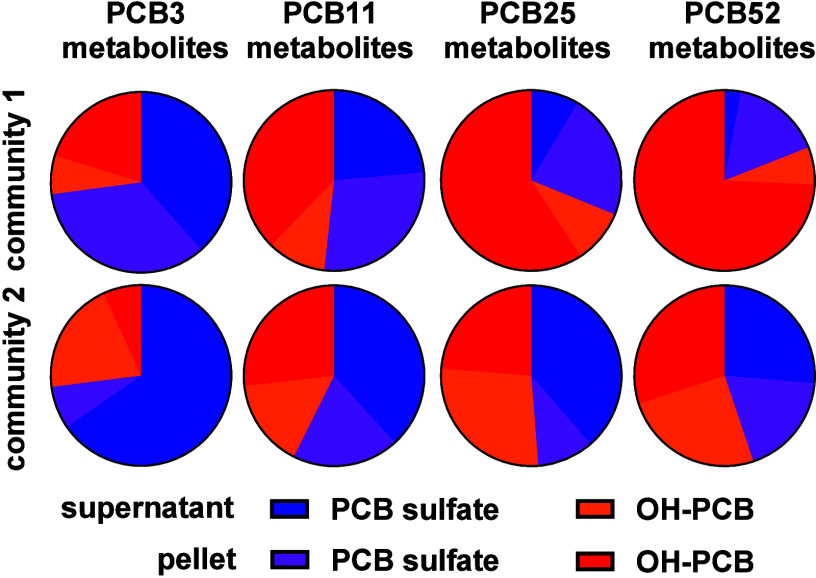
Molar percentage profiles of PCB metabolites (PCB sulfate and corresponding OH-PCB) in the media and pellet after the PCB sulfates were incubated with two human microbial communities.

In conclusion, these findings highlight the influence of microbiome composition, host factors (e.g., sex), and chlorination level on the metabolism of PCB sulfates to OH-PCBs. The observed sex-dependent differences in PCB sulfate metabolism by mouse microbiomes and community-dependent variability in PCB sulfate metabolism by human microbiomes underscore the importance of microbiome diversity in modulating PCB metabolism. Notably, the human microbiome appears to have a pronounced variability in PCB sulfate metabolism, reflecting its broad compositional and functional heterogeneity. In addition to enzymatic metabolism, differences in the protein and lipid composition of gastrointestinal microbiota may influence the partitioning and metabolism of PCBs and OH-PCBs *in vitro* and *in vivo*. These results advance our understanding of the microbial contribution to PCB metabolism and its potential implications for PCB bioavailability, toxicity, and individual susceptibility. Future studies should investigate the specific microbial taxa and enzymatic pathways responsible for these processes to inform personalized approaches for mitigating PCB exposure risks.

## Data Availability

The data are available free of charge on Iowa Research Online at https://doi.org/10.25820/data.007552.

## Supporting Information Available

Description of chemicals, the animal study, metagenomic shotgun sequencing, studies providing a rationale for the PCB sulfate selection, mass spectrometry parameters, and molar percentages and partitioning ratios of PCB metabolites in supernatants and microbial pellets (PDF)

## Abbreviations

OH-PCBs: hydroxylated PCBs
PCBs: polychlorinated biphenyls
