# Supplementary material for: Deconjugation of Polychlorinated Biphenyl Sulfates to Hydroxylated PCBs by Anaerobically Cultured Mouse and Human Gut Microbiota
Source: Chem Res Toxicol. 2025 Mar 25;38(4):557–60. doi: 10.1021/acs.chemrestox.5c00016 (PMC12015953; doi:10.1021/acs.chemrestox.5c00016)
Supplement: Supplementary file 1 — tx5c00016_si_001.pdf [file tx5c00016_si_001.pdf]

Supporting Information

Deconjugation of Polychlorinated Biphenyl Sulfates to Hydroxylated PCBs by Anaerobically Cultured Mouse and Human Gut Microbiota

Xueshu Li[a,‡], Joe J. Lim[b,‡], Cayen Rong[b], Hans-Joachim Lehmler[a,*], Julia Yue Cui[b,*]

[a]Department of Occupational and Environmental Health, University of Iowa, Iowa City, IA 52242, USA. [b]Department of Environmental and Occupational Health Sciences, University of Washington, Seattle, WA 98105, USA

Corresponding Authors

Hans-Joachim Lehmler
Department of Occupational and Environmental Health
University of Iowa
Iowa City, IA 52242
Email: hans-jaochim-lehmler@uiowa.edu
Phone: +1-319-335-4981

Julia Yue Cui
Department of Environmental and Occupational Health Sciences
University of Washington
Seattle, WA 98105
Email: juliacui@uw.edu
Phone: +1-206-616-4331

Table of contents

Chemicals **S3**

Animal study **S3**

Demographics of the human subjects **S3**

Metagenomic shotgun sequencing **S4**

Table S1. Examples of studies investigating the toxicity and occurrence of the PCB metabolites in this study. **S5**

Table S2. Unique identifiers of the analytical PCB and PCB metabolite standards used in this study. **S6**

Table S3. The mass spectrometry parameters and MRM mass transitions for the PCB metabolites analysis. **S8**

Table S4. Molar percentage (%) of PCB metabolites in the supernatant (S) and pellet (P) after PCB sulfates were incubated with microbiomes from male and female germ-free and conventional, respectively. **S9**

Table S5. Partitioning ratios of OH-PCBs and PCB sulfates in supernatant (S) to pellet (P) in experiments using a mouse microbiome. **S10**

Table S6. Partitioning ratios of OH-PCBs and PCB sulfates in supernatant (S) to pellet (P) of pooled microbiomes from de-identified healthy male (community 1, n=2) and female (community 2, n=2). **S11**

Table S7. Molar percentage (%) of PCB metabolites in the supernatant (S) and pellet (P) after PCB sulfates were incubated with pooled microbiomes from de-identified healthy male (community 1, n=2) and female (community 2, n=2). **S12**

References **S13**

Chemicals

Hydroxylated PCBs, including 4'-hydroxy-4-chlorobiphenyl (4'-OH-PCB3), 4-hydroxy-3,3'-dichlorobiphenyl (4-OH-PCB11), 4'-hydroxy-2,3',4-trichlorobiphneyl (4'-OH-PCB25), 4-hydroxy-2,2',5,5'-tetrachlorobiphenyl (4-OH-PCB52), 4'-hydroxy-3-fluoro-4-chlorobiphenyl (3-F, 4'-OH-PCB3), and their corresponding ammonium sulfate salts (i.e., Sulfuric acid mono-(4'-chlorobiphenyl-4-yl) ester, ammonium salt (4'-PCB3 sulfate), sulfuric acid mono-(3,3'-dichlorobiphenyl-4-yl) ester, ammonium salt (4-PCB11 sulfate), sulfuric acid mono-(2',3,4'-trichlorobiphneyl-4-yl) ester, ammonium salt (4'-PCB25 sulfate), sulfuric acid mono-(2,2',5,5'-tetrachlorobiphenyl-4-yl) ester, ammonium salt (4-PCB52 sulfate), sulfuric acid mono-(3'-fluoro-4'-chlorobiphenyl-4-yl) ester, ammonium salt (3-F, 4'-PCB3 sulfate)) were synthesized as described previously.[1-4] For unique identifiers of these chemicals, see Table S2.

Animal study

Banked frozen large intestinal content samples collected from three-months old male and female conventional (CV, with microbiome) and germ-free (GF, without microbiome) mice were used for this study. Mice were housed according to the Association for Assessment and Accreditation of Laboratory Animal Care International guidelines. Mice were housed in standard air-filtered cages using autoclaved bedding (autoclaved Enrich-N'Pure, Andersons, Maumee, Ohio). Mice had ad libitum access to non-acidified autoclaved water, as well as standard rodent chow (LabDiet No. 5021 for breeding pairs or to LabDiet No. 5010 for weaned pups) (LabDiet, St Louis, Missouri). Three-month-old male and female CV and GF mice were euthanized by CO_2 followed by cardiac puncture, and large intestinal contents were immediately frozen on dry ice and stored at -80°C until further analysis. The animal procedures were approved by the Institutional Animal Care and Use Committee (IACUC) at the University of Washington (Protocol # 4339-01). For PCB incubation in an anaerobic chamber, large intestinal contents were diluted to 50 mg/mL in DPBS (Sigma-Aldrich D8537) and a reducing agent; see the manuscript text for more information.

Demographics of the human subjects

Human stool samples (BioIVT, Westbury, NY, USA) were pooled from de-identified healthy individuals. Community 1 included male donors: a 47-year-old Hispanic and a 65-year-old Caucasian. Community 2 consisted of female donors: a 59-year-old Caucasian and a 46-year-old Caucasian.

Metagenomic shotgun sequencing

DNA from large intestinal content was extracted using the EZNA Stool DNA kit (Omega Bio-Tek Inc., Norcross, GA). Shallow shotgun metagenomic sequencing was performed at 2 million reads (Diversigen, New Brighton, MN). DNA sequences were aligned to a curated database containing all representative genomes in RefSeq for bacteria with additional manually curated mouse-specific Metagenomically Assembled Genomes (MAGs) and cell-cultured genomes. Only high-quality MAGs (Completeness > 90% & Contamination < 5% via checkm) were considered. Alignments were made at 97% identity against all reference genomes. Every input sequence was compared to every reference sequence in the Diversigen DivDB-Mouse database using fully gapped alignment with BURST. Ties were broken by minimizing the overall number of unique Operational Taxonomic Units (OTUs). For taxonomy assignment, each input sequence was assigned the lowest common ancestor that was consistent across at least 80% of all reference sequences tied for best hit. Taxonomies are based on the Genome Taxonomy Database (GTDB r95). Samples with fewer than 10,000 sequences were discarded. OTUs accounting for less than one-millionth of all strain-level markers and those with less than 0.01% of their unique genome regions covered (and < 0.1% of the whole genome) at the species level were discarded. Centered-log ratio (CLR) normalized taxonomic abundance between two human donors was compared at the species level. Taxa with greater than 30% difference in CLR abundance were plotted as heatmaps using the R package ComplexHeatmap (v.2.13.1). The raw metagenomic shotgun sequencing data have been deposited with the NCBI raw sequence archive under accession PRJNA1240831.

Table S1. Examples of studies investigating the toxicity and occurrence of the PCB metabolites in this study.

Metabolite	Chemical structures	Reference
4'-OH-PCB3	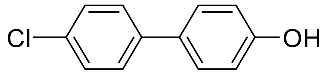	• Toxic in neural cell lines (N27 and SH-SY5Y) and hepatic cell line (HepG2)[5]<br>• Toxic in astrocytes [6]<br>• Showed cardiovascular effect[7]
4-OH-PCB11	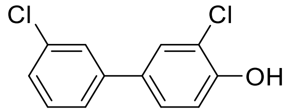	• Toxic in neural cell lines (N27 and SH-SY5Y) and hepatic cell line (HepG2)[5]<br>• Toxic in astrocytes[6]<br>• Toxic in immortalized human prostate epithelial cell[8]<br>• Showed cardiovascular effect[7]<br>• Present in human serum[8]
4'-OH-PCB25	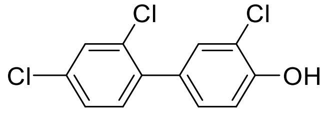	• Toxic in astrocytes[6]<br>• Human metabolite of PCB28[9-11]
4-OH-PCB52	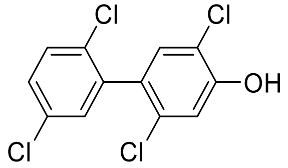	• Toxic in neural cell lines (N27 and SH-SY5Y) and hepatic cell line (HepG2)[5]<br>• Toxic in astrocytes[6,12]<br>• Affects gene expression in human preadipocytes[13]<br>• Showed cardiovascular effect[7]<br>• Metabolite formed by human CYP2A6[14]<br>• Metabolite formed in microsomal metabolism studies[15-18]<br>• Metabolite detected in PCB52 exposed mice[19,20]
4'-PCB3 sulfate	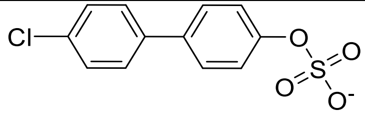	• Detected in urban and rural US populations[21]<br>• Toxic in neural cell lines (N27 and SH-SY5Y) and hepatic cell line (HepG2)[5]<br>• Hydrolyzed to the corresponding OH-PCB by sulfatases present in human hepatic microsomes[22]<br>• Showed cardiovascular effect[7]
4-PCB11 sulfate	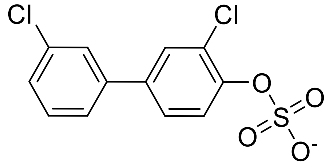	• Detected in human serum[21,23]<br>• Toxic in neural cell lines (N27 and SH-SY5Y) and hepatic cell line (HepG2)[5]<br>• Hydrolyzed to the corresponding OH-PCB by sulfatases present in human hepatic microsomes[22]<br>• Showed cardiovascular effect[7]
4'-PCB25 sulfate	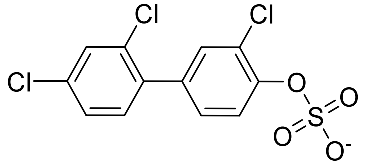	• Detected in urban and rural US populations[21]<br>• Hydrolyzed to the corresponding OH-PCB by sulfatases present in human hepatic microsomes[22]
4-PCB52 sulfate	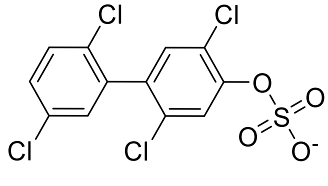	• Detected in urban and rural US populations[21]<br>• Toxic in neural cell lines (N27 and SH-SY5Y) and hepatic cell line (HepG2)[5]<br>• Toxic in astrocytes[6,12]<br>• Hydrolyzed to the corresponding OH-PCB by sulfatases present in human hepatic microsomes[22]<br>• Showed cardiovascular effect[7]

Table S2. Unique identifiers of the analytical PCB and PCB metabolite standards used in this study.

Abbreviation	IUPAC Name	FORMULA	Isomeric SMILES	InChI	InChIKey	CAS Registry Number	CAS Registry URL	PubChem CID	PubChem link	DTXSID	Comptox link
4'-OH-PCB3	4-(4-Chlorophenyl)phenol	C12H9ClO	ClC1=CC=C(C2=CC=C(O)C=C2)C=C1	InChI=1S/C12H9ClO/c13-11-5-1-9(2-6-11)10-3-7-12(14)8-4-10/h1-8,14H	ICVFJPSNAUMFCW-UHFFFAOYSA-N	28034-99-3	https://commonchemistry.cas.org/detail?cas_rn=28034-99-3	91589	https://pubchem.ncbi.nlm.nih.gov/compound/91589	DTXSID5022354	https://comptox.epa.gov/dashboard/DTXSID5022354
4-OH-PCB11	2-chloro-4-(3-chlorophenyl)phenol	C12H8Cl2O	OC(C(Cl)=C1)=CC=C1C2=CC=CC(Cl)=C2	InChI=1S/C12H8Cl2O/c13-10-3-1-2-8(6-10)9-4-5-12(15)11(14)7-9/h1-7,15H	JOHAARQQFBMIOV-UHFFFAOYSA-N	53890-78-1	https://commonchemistry.cas.org/detail?cas_rn=53890-78-1	186674	https://pubchem.ncbi.nlm.nih.gov/compound/186674	DTXSID10202159	https://comptox.epa.gov/dashboard/DTXSID10202159
4'-OH-PCB25	2-chloro-4-(2,4-dichlorophenyl)phenol		OC(C(Cl)=C1)=CC=C1C2=CC=C(Cl)C=C2Cl	InChI=1S/C12H7Cl3O/c13-8-2-3-9(10(14)6-8)7-1-4-12(16)11(15)5-7/h1-6,16H	IPQDZKABLRZERH-UHFFFAOYSA-N	358767-68-7	https://commonchemistry.cas.org/detail?cas_rn=358767-68-7	53221454	https://pubchem.ncbi.nlm.nih.gov/compound/53221454	DTXSID50686095	https://comptox.epa.gov/dashboard/DTXSID50686095
4-OH-PCB52	2,5-dichloro-4-(2,5-dichlorophenyl)phenol	C12H6Cl4O	C1=CC(=C(C=C1Cl)C2=CC(=C(C=C2Cl)O)Cl)Cl	InChI=1S/C12H6Cl4O/c13-6-1-2-9(14)7(3-6)8-4-11(16)12(17)5-10(8)15/h1-5,17H	ZKDSNFDCQYBBIU-UHFFFAOYSA-N	51274-68-1	https://commonchemistry.cas.org/detail?cas_rn=51274-68-1	39971	https://pubchem.ncbi.nlm.nih.gov/compound/39971	DTXSID10199272	https://comptox.epa.gov/dashboard/DTXSID10199272
3-F,4'-OH-PCB3	4-(4-chloro-3-fluorophenyl)phenol	C12H8ClFO	C1=CC(=CC=C1C2=CC(=C(C=C2)Cl)F)O	InChI=1S/C12H8ClFO/c13-11-6-3-9(7-12(11)14)8-1-4-10(15)5-2-8/h1-7,15H	DBNVNCYZHMXJJL-UHFFFAOYSA-N	893736-99-7	https://commonchemistry.cas.org/detail?cas_rn=893736-99-7	20099942	https://pubchem.ncbi.nlm.nih.gov/compound/20099942	DTXSID201279593	https://comptox.epa.gov/dashboard/DTXSID201279593
4'-PCB3 sulfate	4-(4-Chlorophenyl)phenyl ammonium sulfate	C12H12ClNO4S	ClC1=CC=C(C2=CC=C(OS(=O)([O-])=O)C=C2)C=C1.[NH4+]	InChI=1S/C12H9ClO4S.H3N/c13-11-5-1-9(2-6-11)10-3-7-12(8-4-10)17-18(14,15)16;/h1-8H,(H,14,15,16);1H3	CAVDLSYGQKJVAW-UHFFFAOYSA-N	na	na	na	na	na	na
4-PCB11 sulfate	2-chloro-4-(3-chlorophenyl)phenyl ammonium sulfate	C12H11Cl2NO4S	O=S(OC(C=C1)=C(Cl)C=C1C2=CC(Cl)=CC=C2)([O-])=O.[NH4+]	InChI=1S/C12H8Cl2O4S.H3N/c13-10-3-1-2-8(6-10)9-4-5-12(11(14)7-9)18-19(15,16)17;/h1-7H,(H,15,16,17);1H3	RHIHLBMOBKSLHK-UHFFFAOYSA-N	na	na	na	na	na	na

Table S1 (continued). Unique identifiers of the analytical PCB and PCB metabolite standards used in this study.

Abbreviation	IUPAC Name	FORMULA	Isomeric SMILES	InChI	InChIKey	CAS Registry Number	CAS Registry URL	PubChem CID	PubChem link	DTXSID	Comptox link
4'-PCB25 sulfate	2-chloro-4-(2,4-dichlorophenyl)phenyl ammonium sulfate	C12H10Cl3NO4S	O=S(OC(C=C1)=C(Cl)C=C1C2=C(Cl)C=C(Cl)C=C2)([O-])=O.[NH4+]	InChI=1S/C12H7Cl3O4S.H3N/c13-8-2-3-9(10(14)6-8)7-1-4-12(11(15)5-7)19-20(16,17)18;/h1-6H,(H,16,17,18);1H3	GEYOZNBJRMQSLF-UHFFFAOYSA-N	na	na	na	na	na	na
4-PCB52 sulfate	2,5-dichloro-4-(2,5-dichlorophenyl)phenyl ammonium sulfate	C12H9Cl4NO4S	O=S(OC(C=C1Cl)=C(Cl)C=C1C2=C(Cl)C=CC(Cl)=C2)([O-])=O.[NH4+]	InChI=1S/C12H6Cl4O4S.H3N/c13-6-1-2-9(14)7(3-6)8-4-11(16)12(5-10(8)15)20-21(17,18)19;/h1-5H,(H,17,18,19);1H3	DAQCKCZRVSNHHX-UHFFFAOYSA-N	na	na	na	na	na	na
3-F,4'-PCB3 sulfate	4-(3-fluoro-4-chlorophenyl)-phenyl ammonium sulfate	C12H11ClFNO4S	ClC1=C(F)C=C(C2=CC=C(OS(=O)([O-])=O)C=C2)C=C1.[NH4+]	InChI=1S/C12H8ClFO4S.H3N/c13-11-6-3-9(7-12(11)14)8-1-4-10(5-2-8)18-19(15,16)17;/h1-7H,(H,15,16,17);1H3	MUYMCEBMJRPJFT-UHFFFAOYSA-N	na	na	na	na	na	na

Table S3. The mass spectrometry parameters and MRM mass transitions for the PCB metabolites analysis.

**Compound**	**RT (min)**	**Q1 mass (Da)**	**Q3 mass (Da)**	**EP (V)**	**CE (V)**	**CXP (V)**
4'-PCB3 sulfate	2.77	283.0895	202.944	-10	-33	-15
3-F, 4'-PCB3 sulfate	3.12	301.075	220.925	-10	-31	-7
4-PCB11 sulfate	3.93	316.985	236.896	-10	-27	-7
4'-PCB25 sulfate	5.43	350.9206	270.8807	-10	-31	-9
4-PCB52 sulfate	5.59	386.8541	306.8648	-10	-32	-10
4-OH-PCB3	6.27	203.0569	203.057	-10	-5	-1
3-F, 4'-OH-PCB3	6.65	221.046	221.046	-10	-5	-1
4-OH-PCB11	7.61	237.0131	237.013	-10	-5	-1
4'-OH-PCB25	8.56	270.9607	270.961	-10	-5	-1
4-OH-PCB52	8.6	306.9459	306.946	-10	-5	-1

RT, retention time; EP, entrance potential; CE, collision energy; CXP, collision cell exit potential.

Table S4. Molar percentage (%) of PCB metabolites in the supernatant (S) and pellet (P) after PCB sulfates were incubated with microbiomes from male and female germ-free and conventional, respectively.

PCB metabolite	Fraction	M_{GF}	M_{CV}	F_{GF}	F_{CV}
PCB3 sulfate	S	89.0 ± 0.8	84 ± 5	89 ± 1	88 ± 3
	P	10 ± 1	11 ± 2	10 ± 1	9 ± 2
OH-PCB3	S	1.0 ± 0.3	4 ± 3	0.6 ± 0.6	2.3 ± 0.9
	P	0.07 ± 0.04	1.0 ± 0.9	0.10 ± 0.05	0.6 ± 0.4
PCB11 sulfate	S	87 ± 1	64 ± 17	87 ± 1.	79 ± 7
	P	12.4 ± 0.8	9 ± 2	12 ± 2	10 ± 2
OH-PCB11	S	1.0 ± 0.2	19 ± 13	0.7 ± 0.3	8.3 ± 4
	P	0.12 ± 0.05	9 ± 6	0.26 ± 0.02	3 ± 2
PCB25 sulfate	S	81.7 ± 0.9	61 ± 16	82 ± 3	76 ± 7
	P	17 ± 1	12 ± 4	16 ± 3	14 ± 3
OH-PCB25	S	1.2 ± 0.3	17 ± 12	1.3 ± 0.2	7 ± 5
	P	0.08 ± 0.02	11 ± 8	0.12 ± 0.09	4 ± 2
PCB52 sulfate	S	79 ± 2	39 ± 25	80 ± 3	66 ± 13
	P	20 ± 2	10 ± 9	19 ± 2	15 ± 4
OH-PCB52	S	0.42 ± 0.07	28 ± 19	0.7 ± 0.2	11 ± 8
	P	0.02 ± 0.01	24 ± 15	0.06 ± 0.02	8 ± 6

Abbreviations: M_{GF}, male germ-free; M_{CV}, male conventional; F_{GF}, female germ-free; F_{CV}, female conventional.

Table S5. Partitioning ratios of OH-PCBs and PCB sulfates in supernatant (S) to pellet (P) in experiments using a mouse microbiome. Data showed as mean ± SD, n=3 for all groups.

**PCB metabolite**	$\mathbf{M_{GF}}$	$\mathbf{M_{CV}}$	$\mathbf{F_{GF}}$	$\mathbf{F_{CV}}$
PCB3 sulfate	9 ± 1	8 ± 2	9 ± 1	10 ± 2
PCB11 sulfate	7.0 ± 0.5	6.8 ± 0.6	7 ± 1	8 ± 2
PCB25 sulfate	4.8 ± 0.4	5.4 ± 0.5	5 ± 1	6 ± 1
PCB52 sulfate	4.0 ± 0.6	4.1 ± 0.8	4.3 ± 0.7	5 ± 1
OH-PCB3	16 ± 6	15 ± 19	8 ± 8	5 ± 3
OH-PCB11	9 ± 4	2.1 ± 0.3	3 ± 1	2.6 ± 0.6
OH-PCB25	15 ± 7	1.4 ± 0.1	17 ± 15	1.9 ± 0.4
OH-PCB52	26 ± 15	1.1 ± 0.2	13 ± 1	1.6 ± 0.6

Abbreviations: M_{GF}, male germ-free; M_{CV}, male conventional; F_{GF}, female germ-free; F_{CV}, female conventional.

Table S6. Partitioning ratios of OH-PCBs and PCB sulfates in supernatant (S) to pellet (P) of pooled microbiomes from de-identified healthy male (community 1, n=2) and female (community 2, n=2).

PCB metabolite	Community 1	Community 2
PCB3 sulfate	5.5	3.2
PCB11 sulfate	1.7	2.4
PCB25 sulfate	0.9	1.4
PCB52 sulfate	0.4	1.0
OH-PCB3	1.7	1.1
OH-PCB11	0.8	0.7
OH-PCB25	0.4	0.4
OH-PCB52	0.2	0.6

Table S7. Molar percentage (%) of PCB metabolites in the supernatant (S) and pellet (P) after PCB sulfates were incubated with pooled microbiomes from de-identified healthy male (community 1, n=2) and female (community 2, n=2).

PCB metabolite	Fraction	Community 1	Community 2
PCB3 sulfate	S	38.4	65.4
	P	6.9	20.2
OH-PCB3	S	34.5	7.6
	P	20.2	6.8
PCB11 sulfate	S	8.9	38.3
	P	5.2	15.8
OH-PCB11	S	37.3	19.1
	P	48.7	26.8
PCB25 sulfate	S	8.6	38.6
	P	9.7	27.5
OH-PCB25	S	22.5	10.2
	P	59.2	23.7
PCB52 sulfate	S	2.9	26.4
	P	6.7	25.5
OH-PCB52	S	16.1	18.5
	P	74.3	30.0

References

(1) Li, X.; Lehmler, H.-J. Dataset for synthesis and authentication of 2,2',5,5'-tetrachlorobiphenyl-4-ol (4-OH-PCB 52). *University of Iowa (dataset)*, **2022**. https://doi.org/10.25820/data.006178.

(2) Li, X.; Lehmler, H.-J. Dataset for synthesis and authentication of 3,3'-dichlorobiphenyl-4-ol (4-OH-PCB 11). *University of Iowa (dataset)*, **2022**. https://doi.org/10.25820/data.006182.

(3) Dhakal, K.; He, X. R.; Lehmler, H. J.; Teesch, L. M.; Duffel, M. W.; Robertson, L. W. Identification of sulfated metabolites of 4-chlorobiphenyl (PCB3) in the serum and urine of male rats. *Chem Res Toxicol* **2012**, *25* (12), 2796-2804.

(4) Lehmler, H. J.; Robertson, L. W. Synthesis of hydroxylated PCB metabolites with the Suzuki-coupling. *Chemosphere* **2001**, *45* (8), 1119-1127.

(5) Rodriguez, E. A.; Vanle, B. C.; Doorn, J. A.; Lehmler, H. J.; Robertson, L. W.; Duffel, M. W. Hydroxylated and sulfated metabolites of commonly observed airborne polychlorinated biphenyls display selective uptake and toxicity in N27, SH-SY5Y, and HepG2 cells. *Environ Toxicol Pharmacol* **2018**, *62*, 69-78.

(6) Paranjape, N.; Dean, L. E.; Martinez, A.; Tjalkens, R. B.; Lehmler, H. J.; Doorn, J. A. Structure-activity relationship of lower chlorinated biphenyls and their human-relevant metabolites for astrocyte toxicity. *Chem Res Toxicol* **2023**, *36* (6), 971-981.

(7) Grimm, F. A.; Klaren, W. D.; Li, X. S.; Lehmler, H. J.; Karmakar, M.; Robertson, L. W.; Chiu, W. A.; Rusyn, I. Cardiovascular effects of polychlorinated biphenyls and their major metabolites. *Environ Health Persp* **2020**, *128* (7), 77008.

(8) Zhu, Y. M.; Mapuskar, K. A.; Marek, R. F.; Xu, W. J.; Lehmler, H. J.; Robertson, L. W.; Hornbuckle, K. C.; Spitz, D. R.; Aykin-Burns, N. A new player in environmentally induced oxidative stress: polychlorinated biphenyl congener, 3,3-dichlorobiphenyl (PCB11). *Toxicol Sci* **2013**, *136* (1), 39-50.

(9) Randerath, I.; Quinete, N.; Müller, J. P.; Stingl, J.; Bertram, J.; Schettgen, T.; Kraus, T.; Ziegler, P. Partial dechlorination of 2,4,4′-trichlorobiphenyl (PCB 28) mediated by recombinant human CYP1A2. *Arch Toxicol* **2024**, *98* (1), 159-163.

(10) Quinete, N.; Esser, A.; Kraus, T.; Schettgen, T. PCB 28 metabolites elimination kinetics in human plasma on a real case scenario: Study of hydroxylated polychlorinated biphenyl (OH-PCB) metabolites of PCB 28 in a highly exposed German Cohort. *Toxicol Lett* **2017**, *276*, 100-107.

(11) Randerath, I.; Schettgen, T.; Mueller, J. P.; Rengelshausen, J.; Ziegler, S.; Quinete, N.; Bertram, J.; Laieb, S.; Schaeffeler, E.; Kaifie, A.; et al. Metabolic activation of WHO-congeners PCB28, 52, and 101 by human CYP2A6: evidence from in vitro and in vivo experiments. *Arch Toxicol* **2024**, *98* (11), 3739-3753.

(12) Paranjape, N.; Strack, S.; Lehmler, H. J.; Doorn, J. A. Astrocyte mitochondria are a sensitive target of PCB52 and its human-relevant metabolites. *ACS Chem Neurosci* **2024**, *15* (15), 2729-2740.

(13) Gourronc, F. A.; Chimenti, M. S.; Lehmler, H. J.; Ankrum, J. A.; Klingelhutz, A. J. Hydroxylation markedly alters how the polychlorinated biphenyl (PCB) congener, PCB52, affects gene expression in human preadipocytes. *Toxicol in Vitro* **2023**, *89*, 105568.

(14) Shimada, T.; Kakimoto, K.; Takenaka, S.; Koga, N.; Uehara, S.; Murayama, N.; Yamazaki, H.; Kim, D.; Guengerich, F. P.; Komori, M. Roles of human CYP2A6 and monkey CYP2A24 and 2A26 cytochrome P450 enzymes in the oxidation of 2,5,29,59-tetrachlorobiphenyl. *Drug Meta Dispos* **2016**, *44* (12), 1899-1909.

(15) Borlakoglu, J. T.; Haegele, K. D.; Reich, H. J.; Dils, R. R.; Wilkins, J. P. G. *In vitro* metabolism of [^{14}C]4-chlorobiphenyl and [^{14}C]2,2',5,5'-tetrachlorobiphenyl by hepatic microsomes from rats and pigeons - evidence against an obligatory arene oxide in aromatic hydroxylation reactions. *Int J Biochem* **1991**, *23* (12), 1427-1437.

(16) Forgue, S. T.; Allen, J. R. Identification of an arene oxide metabolite of 2,2',5,5'-tetrachlorobiphenyl by gas chromatography-mass spectroscopy. *Chem-Biol Interact* **1982**, *40* (2), 233-245.

(17) Preston, B. D.; Miller, J. A.; Miller, E. C. Nonarene oxide aromatic ring hydroxylation of 2,2',5,5'-tetrachlorobiphenyl as the major metabolic pathway catalyzed by phenobarbital-Induced rat-liver microsomes. *J Biol Chem* **1983**, *258* (13), 8304-8311.

(18) de Bruin, O. M.; Birnboim, H. C. A method for assessing efficiency of bacterial cell disruption and DNA release. *BMC Microbiology* **2016**, *16*, 197.

(19) Bullert, A. J.; Li, X. S.; Gautam, B.; Wang, H.; Adamcakova-Dodd, A.; Wang, K.; Thorne, P. S.; Lehmler, H. J. Distribution of 2,2′,5,5′-tetrachlorobiphenyl (PCB52) metabolites in adolescent rats after acute nose-only inhalation exposure. *Environ Sci Tech* **2024**, *58* (14), 6105-6116.

(20) Bullert, A.; Li, X. S.; Zhang, C. Y.; Lee, K.; Pulliam, C. F.; Cagle, B. S.; Doorn, J. A.; Klingelhutz, A. J.; Robertson, L. W.; Lehmler, H. J. Disposition and metabolomic effects

of 2,2′,5,5′-tetrachlorobiphenyl in female rats following intraperitoneal exposure. *Environ Toxicol Pharmacol* **2023**, *102*, 104245.

(21) Zhang, D.; Saktrakulkla, P.; Marek, R. F.; Lehmler, H. J.; Wang, K.; Thorne, P. S.; Hornbuckle, K. C.; Duffel, M. W. PCB sulfates in serum from mothers and children in urban and rural US communities. *Environ Sci Tech* **2022**, *56* (10), 6537-6547.

(22) Duffel, M. W.; Tuttle, K.; Lehmler, H. J.; Robertson, L. W. Human hepatic microsomal sulfatase catalyzes the hydrolysis of polychlorinated biphenyl sulfates: A potential mechanism for retention of hydroxylated PCBs. *Environ Toxicol Pharmacol* **2021**, *88*, 103757.

(23) Grimm, F. A.; Lehmler, H. J.; Koh, W. X.; DeWall, J.; Teesch, L. M.; Hornbuckle, K. C.; Thorne, P. S.; Robertson, L. W.; Duffel, M. W. Identification of a sulfate metabolite of PCB 11 in human serum. *Environ Int* **2017**, *98*, 120-128.
